# Co-Formulants in Glyphosate-Based Herbicides Disrupt Aromatase Activity in Human Cells below Toxic Levels

**DOI:** 10.3390/ijerph13030264

**Published:** 2016-02-26

**Authors:** Nicolas Defarge, Eszter Takács, Verónica Laura Lozano, Robin Mesnage, Joël Spiroux de Vendômois, Gilles-Eric Séralini, András Székács

**Affiliations:** 1 Institute of Biology, University of Caen Normandy, EA2608 and Network on Risks, Quality and Sustainable Environment MRSH, Esplanade de la Paix, CS 14032, Caen Cedex 5, France; nicolas.defarge@gmail.com (N.D.); veritol77@gmail.com (V.L.L.); robinmes@gmail.com (R.M.); 2 Agro-Environmental Research Institute, National Agricultural Research and Innovation Centre, H-1022, Herman Ottó u. 15, Budapest, Hungary; e.takacs@cfri.hu (E.T.); a.szekacs@cfri.hu (A.S.); 3 CRIIGEN, 81 rue Monceau, 75008 Paris, France; jmspiroux@wanadoo.fr

**Keywords:** glyphosate-based herbicide, JEG3 cells, endocrine disruption, aromatase, co-formulant, pesticide

## Abstract

Pesticide formulations contain declared active ingredients and co-formulants presented as inert and confidential compounds. We tested the endocrine disruption of co-formulants in six glyphosate-based herbicides (GBH), the most used pesticides worldwide. All co-formulants and formulations were comparably cytotoxic well below the agricultural dilution of 1% (18–2000 times for co-formulants, 8–141 times for formulations), and not the declared active ingredient glyphosate (G) alone. The endocrine-disrupting effects of all these compounds were measured on aromatase activity, a key enzyme in the balance of sex hormones, below the toxicity threshold. Aromatase activity was decreased both by the co-formulants alone (polyethoxylated tallow amine—POEA and alkyl polyglucoside—APG) and by the formulations, from concentrations 800 times lower than the agricultural dilutions; while G exerted an effect only at 1/3 of the agricultural dilution. It was demonstrated for the first time that endocrine disruption by GBH could not only be due to the declared active ingredient but also to co-formulants. These results could explain numerous *in vivo* results with GBHs not seen with G alone; moreover, they challenge the relevance of the acceptable daily intake (ADI) value for GBHs exposures, currently calculated from toxicity tests of the declared active ingredient alone.

## 1. Introduction

Reproductive health is influenced by environmental toxicants, including pesticides [1,2,3]. The declared active ingredients (dAI) of pesticide formulations are not applied in their isolated form in agricultural use. Other substances (co-formulants) are also added, in order to modify the physico-chemical properties or to improve penetration [4] or stability [5,6] of the dAIs. The identity of the co-formulants (declared as inert) is generally kept confidential. Moreover, they are not used in medium or long term *in vivo* toxicity tests of pesticides on mammals for the establishment of their acceptable daily intake (ADI). However, pesticide formulations and co-formulants alone have been demonstrated to be up to 1000 times more toxic to human cells than their dAI at 24 h exposure [7,8]. 

Among pesticides, glyphosate-based herbicides (GBHs), such as Roundup (R), are the most frequently used worldwide, and their residues are common contaminants of ground and surface water [9] and in food and feed [10]. This is partly due to pre-harvest desiccation treatment of non-transgenic cereals with GBHs [10], but mostly because they are sprayed on the 80% of genetically modified edible plants purposely designed to tolerate R [11] and, thus, contain its residues [12]. When other pesticides are generally allowed in edible plants at levels around 0.01–0.1 ppb [13], glyphosate and its metabolite AMPA have among the highest maximum residue limits (MRL), with up to 500 ppm authorized in some genetically modified feed [14]. It is well known that GBHs and their co-formulants, such as polyethoxylated tallow amine (POEA), are more toxic than glyphosate (G) alone, both in *in vitro* [15,16,17,18,19] and *in vivo* studies [20,21,22,23]. Thus, co-formulants may contribute to reproductive side-effects [24,25,26,27,28,29]. Co-formulants added to GBHs in formulation differ between countries and manufacturers. As a result of this variability in co-formulants, and since most of them are not compulsorily declared, the formulation and the dAI are often treated as the same substance, and co-formulants are not target ingredients in (eco)toxicological studies [30,31,32,33,34]. This results in a misconsideration of GBH toxicities in the literature, where different biological effects of formulations due to different co-formulants are possible.

The objective of this work is to test the cellular endocrine disrupting effects of co-formulants in GBHs below toxic levels, both alone and in formulations (mixtures of co-formulants and active ingredient), in comparison to G. Specifically, five co-formulants and six commercial formulations of GBHs were selected (Table 1). For that purpose, the steroidogenic enzyme aromatase, responsible for the irreversible bioconversion of androgens into estrogens [35], was chosen as a target. Aromatase activity was measured by tritiated water release in human cells, a validated model for the assessment of endocrine disrupting effects [36]. It is already known that R inhibits aromatase activity in the human placenta and in JEG3 human placental choriocarcinoma cells [37,38]. JEG3 cells used here are well-characterized and validated as useful models to test toxicities of pesticides [39], corresponding to what is observed in fresh tissue or primary cells [37]. They are even in some instances less sensitive than primary cells [40] and therefore do not overestimate cellular toxicity. To test the toxicity threshold above endocrine disruption, we assayed their mitochondrial succinate dehydrogenase (SD) activity and membrane integrity after 24 h exposure [41].

## 2. Experimental Section 

### 2.1. Chemicals

The 13 xenobiotics investigated were glyphosate (G), G-based herbicides (GBHs), and their putative or known co-formulants; they are reported in Table 1. G (N-phosphonomethyl glycine, G, CAS No: 1071-83-6) was tested in form of the isopropyl ammonium salt (IPA, CAS No: 386411-94-0), the form of the active ingredient found in most GBHs tested in this study; it was obtained from Lamberti S.p.A, Abizzate, Italy. Co-formulants were: (*i*) polyethoxylated tallow amine pure (POEA) with an average ethoxylation rate of 15 (POE-15, CAS No: 61791-26-2, trade name Emulson AG GPE 3SS obtained from Lamberti S.p.A., Abizzate, Italy), and formulated (POEA/F, CAS No: 61791-26-2, trade name Emulson AG GPE 3/SSM obtained from Lamberti S.p.A, Abizzate, Italy) form containing 70% of POE-15; (*ii*) alkyl polyglucoside (APG, CAS No: 383178-66-3/110615-47-9, trade name Plantapon LGC obtained from The Soap Kitchen, Torrington, United Kingdom); (*iii*) POE-APE which is a mixture of alkyl (C8–10) polyoxyethylene ether phosphates (CAS No: 68130-47-2) and polyoxyethylene alkyl ether phosphate (CAS No: 50769-39-6), Trade name Rolfen Bio obtained from Lamberti S.p.A, Abizzate, Italy); and (*iv*) quaternary ammonium compound (QAC, CAS No: 66455-29-6, trade name Emulson AG CB 30 obtained from Lamberti S.p.A, Abizzate, Italy). Co-formulant of Medallon Premium herbicide, APG (alkyl polyglycoside, CAS No: 68515-73-1) is a mixture of D-glucopyrannose linked to different fatty alcohols with chain lengths ranging from C8 to C10 alkylpolyglycoside, and oligomers. This co-formulant was not available for research purposes; thus, we assessed the closest APG available (alkyl polyglycoside, CAS No 110615-49-9, trade name Plantapon LGC) also formed from glucopyrannose, but linked to fatty alcohols with longer chain (C10–18), considered as having comparable toxicological properties [42]. The chemical structures of co-formulants are reported in Table 2.

Formulations were commercially available Glyfos (Cheminova, Hungary, approval 02.5/12019-2/2010), Kapazin (Arysta, Hungary, approval 02.5/12062-2/2010), Medallon Premium (Syngenta, Hungary, approval 02.5/10506-2/2010), Roundup Classic (MON2139, Monsanto, Hungary, approval 02.5/915/2/2010), Total (Sinon Corporation, approval 02.5/12059-2/2010), Roundup WeatherMAX (Monsanto Canada, approval 27487). 3-(4,5-Dimethylthiazol-2-yl)-2,5-diphenyl tetrazolium bromide (MTT) was prepared as a 5 mg/mL stock solution in phosphate-buffered saline (PBS), filtered through a 0.22 µm filter before use, and diluted to 1 mg/mL in a serum-free medium. MTT, 4-androstene-3,17-dione and formestane (4-hydroxyandrost-4-ene-3,17-dione, CGP-32349) were obtained from DM Labo (Caen, France). [1β-^3^H] Androstenedione (specific activity, 25.3 Ci/mmol; 958.3 GBq/mmol) was purchased from DuPont-New England Nuclear (Les Ulis, France). Ultima-Gold LLT was obtained from Perkin-Elmer.

### 2.2. Cell Lines and Treatments

JEG3 cell line (ECACC 92120308) was provided by CERDIC (Sophia-Antipolis, Valbonne, France). Cells were grown in phenol red-free Eagle’s minimum essential medium (EMEM) (Abcys, Paris, France) containing 2 mM glutamine, 1% non-essential amino acid, 100 U/mL of antibiotics (a mixture of penicillin, streptomycin, and fungizone) (Lonza, Saint Beauzire, France), 10 mg/mL of liquid kanamycin (Dominique Dutscher, Brumath, France) and 10% fetal bovine serum (PAA, les Mureaux, France). JEG3 cells were supplemented with 1 mM sodium pyruvate. Cells were grown with this medium at 37 °C (5% CO_2_, 95% air) during 48 h to 80% confluence, then washed and exposed 24 h with serum-free EMEM to G, GBH formulations and their co-formulants. Before treatment, all the G, GBH, and co-formulants were diluted in serum-free medium and adjusted to a similar pH. This model has been previously validated [15], in that toxic effects were similar in presence of serum but delayed by 48 h.

### 2.3. Cell Treatments and Cytotoxicity Biomarkers

Cells upon 80% of confluence were washed with serum-free EMEM and then exposed to various concentrations of G, GBHs, and co-formulants in EMEM serum-free medium for 24 h. After treatments, a succinate dehydrogenase (SD) activity assay (MTT) [41] was applied, as described previously [16]. The integrity of mitochondrial dehydrogenase enzymes indirectly reflects cellular mitochondrial respiration. The optical density was measured at 570 nm using a Mithras LB 940 luminometer (Berthold, Thoiry, France). The bioluminescent Toxilight bioassay (Lonza, Saint Beauzire, France) was applied for the membrane degradation assessment by intracellular adenylate kinase (AK) release in the medium that is described as a necrosis marker [43]. The same luminometer was used to measure luminescence in the methods previously described [16].

### 2.4. Aromatase Activity Measurement

Aromatase activity was evaluated according to the tritiated water release assay [44], with a slight modification as previously described [45]. This method is based on the stereo-specific release of 1β-hydrogen from the androstenedione substrate, which forms tritiated water during aromatization. JEG3 cells were exposed for 22 h at 37 °C (5% CO_2_, 95% air) to 700 μL of non-toxic doses of different xenobiotics. Formestane, a well-known aromatase inhibitor, was used as a positive control. Then 50 μL of 200 nM [1β-^3^H] androstenedione was added, and incubation went on for 120 min more. The reaction was stopped by placing the plates at 4 °C for 10 min. Cell fragments were removed by 5 min centrifugation at 2000 rpm at 4 °C and by addition of 1 mL of chloroform to the 500 μL supernatant. After 5 min centrifugation at 4000 rpm at 4 °C, 0.5 mL of charcoal/dextran (0.25%/0.025%) was added. The mixture was gently agitated, rested at 4 °C for 10 min, and centrifuged at 4000 rpm for 10 min at 4 °C. Supernatant fractions (500 μL) were harvested in 6 mL vials (Pico Prias vial 2000) and 4 mL Ultima Gold LLT was added. The mixture was assessed for radioactivity by a double 5 min scintillation counting (Packard, liquid scintillation counter TRI-CARB 2100TR, USA).

### 2.5. Statistical Analysis

The experiments were repeated at least in triplicate in different weeks on three independent cultures. All data were presented as the means ± standard error of the mean (SEM). In MTT assays, LC_50_ values were the best-fitted value of a non-linear regression using asymmetric (5-parameters) equation with GraphPad Prism 5 (GraphPad software, La Jolla, CA, USA). Statistical differences were determined by a non-parametric Wilcoxon (Mann–Whitney) rank-sum test or, in case of more than two samples, a non-parametric Kruskal–Wallis test followed by a Dunn’s *post hoc* test for multiple comparisons, using GraphPad Prism 5 (GraphPad software, La Jolla, CA, USA). Significant levels were reported with *p* < 0.05 (*), *p* < 0.01 (**) and *p* < 0.001 (***).

## 3. Results 

The cytotoxic effects of co-formulants and formulations in comparison to the declared active ingredient glyphosate were measured by determining mitochondrial respiration and, for the first time, the comparative endocrine disrupting effects of these compounds through aromatase activity inhibition.

### 3.1. Toxicity Thresholds

**Figure 1:**
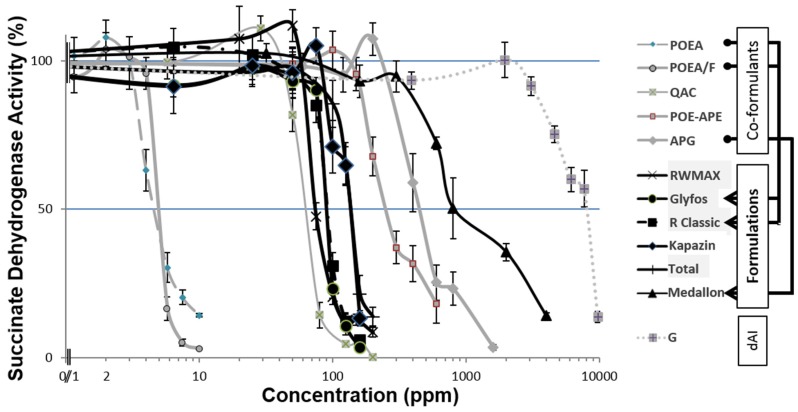
Dose-dependent cytotoxic effects of various glyphosate-based herbicide formulations or co-formulants or the declared active ingredient (dAI) glyphosate alone (G) in the JEG3 human cell line.

For each product, a dose-response curve was determined by MTT test using eight concentrations (Figure 1). LC_50_ values (in ppm *i.e*., mg/L) were calculated (Table 1) by non-linear regression using an asymmetric (5-parameter logistic) equation. The lowest concentration exerting a significant toxic effect (LOEC: Lowest Observed Effect Concentration) was considered to be the toxicity threshold. The highest concentration without significant cytotoxic effect (NOEC: No Observed Effect Concentration) was also reported. For all the products the NOECs were between 6 to 100% below the LOECs and between 15% to 154% below the LC_50_. All the co-formulants and GBHs tested were more toxic than G alone; furthermore, 2 co-formulants showed higher cytotoxicity than the corresponding formulation. POEA (pure and formulated) and QAC were clearly the most toxic, respectively 2000 and 136 times more than G at their LC_50_ values. POEA was toxic at a dilution of 0.002%, *i.e*., 500–1000 times less than the recommended agricultural dosages of its formulation R Classic (1%–2%). They were followed by POE-APE, and APG was found to be the least toxic. Among the formulations the toxicity order was as follows: Roundup WeatherMAX was the most toxic (LC_50_: 71 ppm), Glyfos (LC_50_: 86 ppm), equivalent to R Classic (LC_50_: 89 ppm), followed by Total (LC_50_: 128 ppm), Kapazin (LC_50_: 130 ppm); and Medallon, with the latter 10 times less toxic than other formulations (LC_50_: 1268 ppm). Difference in toxicity of GBHs presumably occurred due to the type of co-formulant because G concentration was the same in five of the six GBHs tested in this study. Medallon was by far the least toxic; however, its LC_50_ was still around 10 times lower than the recommended agricultural dose, and even this formulation was six times more toxic than G alone.

Effects on the mitochondrial succinate dehydrogenase (SD) activity reflecting cell respiration are shown in comparison to the control (100%) in serum-free medium after 24 h exposure. Concentrations are in ppm in logarithmic scale (agricultural recommended dilution 10,000 ppm). All data are mean ± standard error of the mean (SEM). All the experiments were repeated three times in triplicate. Co-formulants and formulations are indicated on the right. G is isopropyl ammonium salt of glyphosate. RWMAX: Roundup WeatherMAX.

### 3.2. Comparative Toxicities within and between Formulations

**Figure 2:**
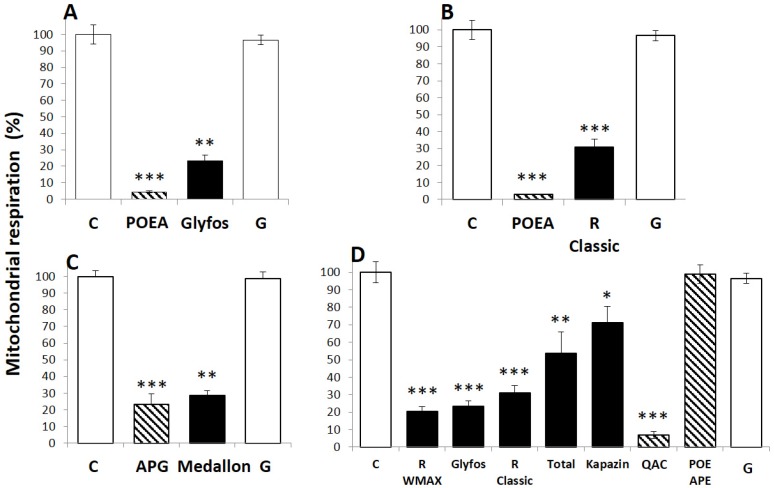
Inhibition of cell respiration by co-formulants and formulations at similar levels. * *p* < 0.05, ** *p* < 0.01, *** *p* < 0.001. (**A**) 100 ppm of Glyfos is compared to the content in POEA (10 ppm) and G (48.6 ppm); (**B**) 100 ppm of Glyfos is compared to the content in POEA (10 ppm) and G (48.6 ppm); (**C**) 2000 ppm of Medallon is compared to the content in APG (800 ppm) and G (975 ppm); (**D**) 100 ppm of the formulations are compared to the content in G (48.6 ppm). Two co-formulants were also tested around their LC_50_ (QAC, 58 ppm; POE-APE, 222 ppm, see Table 1).

**Figure 3:**
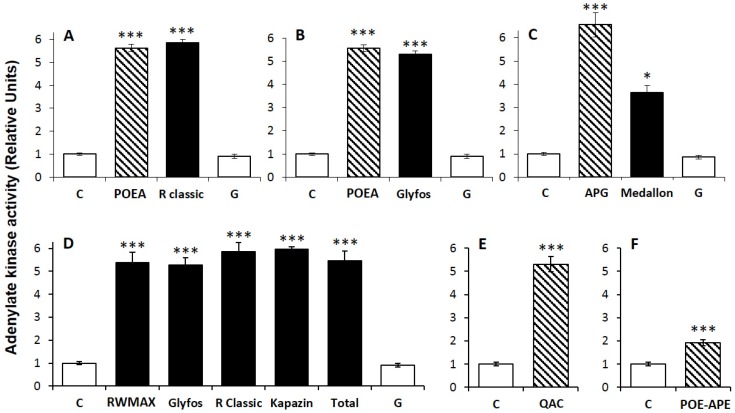
Membrane integrity disruption by formulations and co-formulants. * *p* < 0.05, ** *p* < 0.01, *** *p* < 0.001. (**A**) 100 ppm of Glyfos is compared to the content in POEA (10 ppm) and G (48.6 ppm); (**B**) 100 ppm of Glyfos is compared to the content in POEA (10 ppm) and G (48.6 ppm); (**C**) 2000 ppm of Medallon is compared to the content in APG (800 ppm) and G (975 ppm); (**D**) 100 ppm of the formulations are compared to the content in G (48.6 ppm); Two co-formulants were also tested around their LC_50_ (see Table 1): (**E**) QAC, 58 ppm; (**F**) POE-APE, 222 ppm.

In three of the formulations tested (Glyfos, R Classic and Medallon), the main co-formulant and its concentration is declared, in addition to the content in G (Table 1), allowing a direct comparison (Figure 2). For each formulation, the level tested was chosen around the LC_50_ (for instance, for Glyfos and R Classic, the level tested was 100 ppm, which is 100 times less than the lowest agricultural dose; LC_50_ values are 86 and 89 ppm, respectively), and compared to the amount of the known corresponding co-formulant and of G that it contains. For the three formulations (Figure 2A–C), comparable toxicities of the declared co-formulants and corresponding formulations were evidenced, and none with G, even if the formulations, containing several other co-formulants, showed slightly lower toxicity than the main co-formulant declared. In fact, G alone induced neither inhibition of mitochondrial respiration at this level, nor disruption of the cell membrane, in contrast to the corresponding co-formulants (Figure 3). Two other pesticide co-formulants, QAC and POE-APE, not declared in these formulations, were also investigated, and they effectively present LC_50_ values 200 to 50 times lower than G alone, respectively, They are, thus, far more toxic than G and show a general mechanism of co-formulant effects, even if they have differential toxicities (Figure 2D). When formulations are compared at the same dose far below agricultural dilutions (at 100 ppm, Figure 2D), and also compared to the 100 ppm of two other pesticide co-formulants, QAC and POE-APE, not declared in these formulations, a hierarchy of toxicity is observed: the co-formulant QAC and the formulations are far more cytotoxic than G (135 times for QAC and between 61 to 111 times for the formulations). Even POE-APE, not cytotoxic at 100 ppm, remains 19 times more toxic than G. Whatever co-formulants the formulations contain, none of them are inert in human cells. Indeed, all the co-formulants alone and in formulation triggered membrane disruption (Figure 3), but G alone did not do so at these levels.

Mitochondrial succinate dehydrogenase (SD) activities of JEG3 cells (% of the control values) were measured after 24 h exposure to formulations of glyphosate-based herbicides (GBH), their declared co-formulants alone or glyphosate (G) alone as following: A. The concentrations of POEA (9 ppm) and G (48.6 ppm) are those present in 100 ppm of Glyfos. B. The concentrations of POEA (18 ppm) and G (48.6 ppm) are those present in 100 ppm of R Classic. C. The concentrations of APG (800 ppm) and G (975 ppm) are those present in 2000 ppm of Medallon. D. 100 ppm of the formulations (R WeatherMAX – R WMAX –, Glyfos, R Classic, Kapazin, and Total) are compared to 100 ppm of two co-formulants (QAC and POE-APE) and glyphosate alone (G). All data are the mean ± standard error of the mean (SEM). All the experiments were repeated three times in triplicate. Statistically significant differences from the controls were determined by a Kruskal-Wallis nonparametric test followed by a *post hoc* test using significant levels of 0.05 (*), 0.01 (**) and 0.001 (***). G is isopropyl ammonium salt of glyphosate. C: Control; R: Roundup.

Loss of cell membrane integrity reflecting necrosis was measured by adenylate kinase activity corresponding to a leakage (active in the medium) expressed in relative units after 24 h of treatments in JEG3 cells after treatments described in Figure 1. Data are the mean ± standard error of the mean (SEM). All the experiments were repeated three times in triplicate. Statistically significant differences from the controls were determined by a Kruskal-Wallis non-parametric test using significant levels of 0.05 (*), 0.01 (**) and 0.001 (***). G is isopropyl ammonium salt of glyphosate. C: Control; R: Roundup.

### 3.3. Aromatase Inhibition by Co-Formulants within and between Formulations

**Figure 4:**
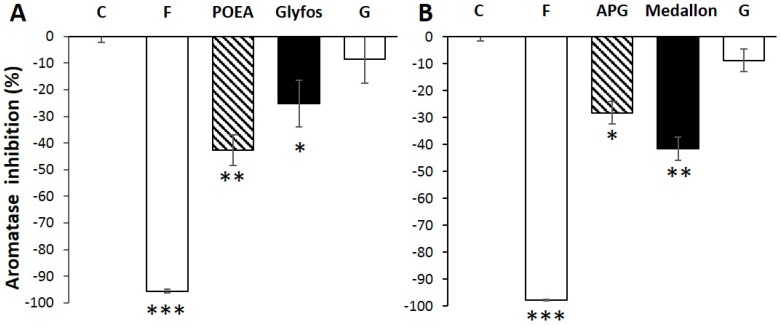
Aromatase inhibition by formulations and their co-formulants alone at similar levels. * *p* < 0.05, ** *p* < 0.01, *** *p* < 0.001. (**A**) 25 ppm of Glyfos is compared to the content in POEA (2.5 ppm) and glyphosate (16 ppm); (**B**) 300 ppm of Medallon is compared to the content in APG (120 ppm) and glyphosate (146 ppm). These concentrations are 1.2–2 times below the NOEC and 1.4–3.4 below the toxicity threshold (LOEC).

Aromatase inhibition was tested at concentrations between 1.2 to three times below the NOEC and 1.2 to two times below the toxic level (significant toxicity threshold, LOEC), in order to avoid measuring the general toxicological effect. Moreover, these differences of thresholds corresponded to the differential effects between co-formulants and formulations (see Section 3.2). Aromatase assays were performed at 25 ppm of Glyfos (containing 2.5 and 16 ppm of POEA and G, respectively), and 300 ppm of Medallon (containing 120 ppm of APG, its co-formulant, and 190 ppm of G). These concentrations are between 1.4–3.4 times below the toxicity thresholds (LOECs). Concerning Glyfos (Figure 4A), aromatase activity was decreased by the co-formulant alone (POEA, −43%, *p* < 0.01) and slightly by the formulation (−25%, *p* < 0.05), as a mixture of co-formulant and dAI. G alone at the same level as in the formulation is unable to have any significant effect. Formestane (4-hydroxyandrost-4-ene-3,17-dione), a known aromatase inhibitor, was used in both cases as a positive control to demonstrate the specificity of the effect. For Medallon (Figure 4B), the co-formulant (APG; −28% to control, *p* < 0.05) was less endocrine-disrupting than the formulation (−42%, *p* < 0.01), but this was not due to G alone, which had no significant effect by itself. This may be due to other non-declared components. Thus, for the first time we demonstrated that the endocrine disruption of these pesticides could not only be due to the declared active ingredient, but also to co-formulants. However, at very high doses of 3000 ppm (1/3 of agricultural dilutions) the declared active ingredient G provoked a significant aromatase inhibition (−51%, *p* < 0.001, data not shown). Nonetheless, this is only theoretical, because at such levels all formulations and co-formulants are lethal in the cells studied.

Viability was always 100% at any of these levels, as measured by the mitochondrial succinate dehydrogenase activity. JEG3 cells were incubated 24 h with a non-cytotoxic dose of the compounds described of the formulations. Formestane (F), a well-known aromatase inhibitor, was used as a positive control. Results are presented as % inhibition of the controls. All data are the mean ± standard error of the mean (SEM). All the experiments were repeated three times in triplicate. Statistically significant differences from the controls (*) were determined by a Kruskal–Wallis nonparametric test followed by a *post hoc* test using significant levels of 0.05 (*), 0.01 (**) and 0.001 (***). G is isopropyl ammonium salt of glyphosate. LOEC: Lowest Observed Effect Concentration; NOEC: No Observed Effect Concentration; C: Control; R: Roundup.

**Figure 5:**
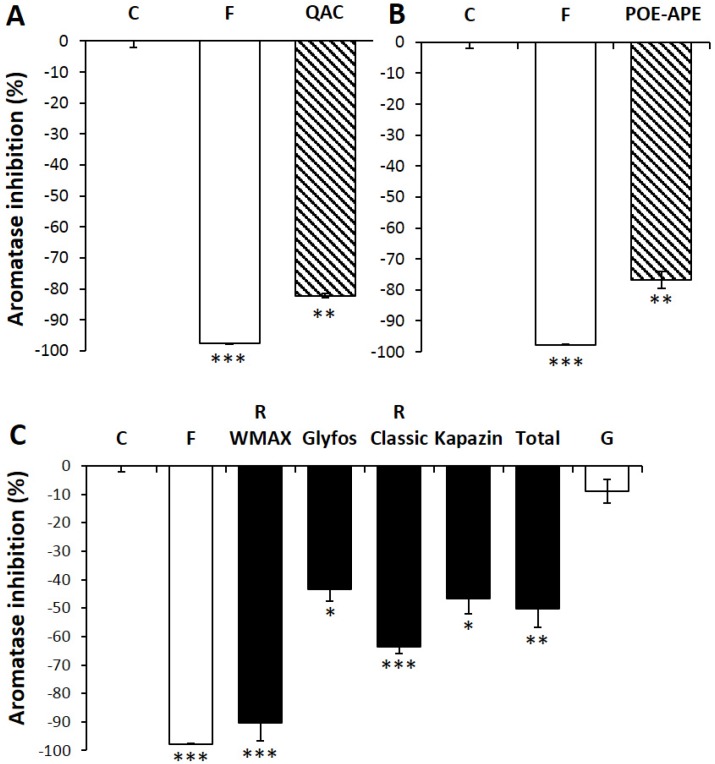
Inhibition of aromatase by co-formulants and formulations. * *p* < 0.05, ** *p* < 0.01, *** *p* < 0.001. (**A**) 25 ppm of the co-formulant QAC is compared to the negative control (C) and to the positive control formestane (F); (**B**) 100 ppm of the co-formulant POE-APE is compared to the negative control (C) and to the positive control formestane (F); (**C**) 50 ppm of formulations of glyphosate-based herbicides R WeatherMAX—R WMAX—Glyfos, R Classic, Kapazin and Total is compared to the negative control (C) and to the positive control formestane (F). Glyphosate alone (isopropyl ammonium salt form) was also tested at its dose present in the formulations (24.3 ppm).

Two other pesticide co-formulants, QAC and POE-APE, not declared in these formulations were, again, studied, and they effectively inhibited aromatase by around 80% (*p* < 0.001; Figure 5A,B). This is, of course, under the NOEC (respectively 1.4 and 1.5 times below, and half of the toxicity threshold for each). This could indicate a common mechanism of co-formulant effects. When formulations are compared at the same dose far below agricultural dilutions (at 50 ppm, 1.2–2 times below the NOEC, *i.e*., 1.4–2.5 times below the LOEC; Figure 5C), a hierarchy of endocrine disruption is observed, and not for G alone, which is inefficient under these conditions. In contrast, POEA and all other co-formulants contained in the formulations are not inert in human cells.

The viability was always 100% at any of these levels, as measured by the mitochondrial succinate dehydrogenase activity. JEG3 cells were incubated 24 h with non-cytotoxic doses of co-formulants or formulations. Formestane (F), a well-known aromatase inhibitor, was used as a positive control. Results are presented as % inhibition of the controls. All data are the mean ± standard error of the mean (SEM). All the experiments were repeated three times in triplicate. Statistically significant differences from the controls were determined by a Kruskal–Wallis nonparametric test followed by a *post hoc* test using significant levels of 0.05 (*), 0.01 (**) and 0.001 (***). C: Control; R: Roundup.

## 4. Discussion

Most co-formulants belong to chemical families of detergents with different chain lengths [8]. Indeed, their CAS numbers refer to classes with common chemical structures rather than single components (Table 2). They are considered as inert and are often kept confidential by manufacturers. 

In this study we measured the differential toxicity of the declared active ingredient and the co-formulants of various GBHs, the major herbicides in the world. Experiments were performed after 24 h exposure on JEG3 human placental cells. Briefly, all co-formulants inhibited aromatase and disrupted mitochondrial respiration (and membranes) at higher concentrations. APG and POEA were 15–18 times and 1200–2000 times more cytotoxic than G, respectively.

Endocrine disrupting effects were measured 1.4–3.3 times below toxicity thresholds, always at levels at least 33 times below the agricultural dilutions. This provides a clearer understanding of pesticide side-effects in formulations. All co-formulants and formulations were cytotoxic at concentrations well below (18–2000 times among co-formulants, 8–141 times among formulations) the recommended agricultural dilution of 1%, and even below any detectable direct toxic effect, they acted as endocrine disrupters. POEA, the declared co-formulant in R Classic and Glyfos, was the most toxic, followed by QAC, an ionic co-formulant. Co-formulant APG contained in Medallon was also shown to be more cytotoxic than the formulation itself, which compensated for its effect. This could be a common feature among pesticide co-formulants, because they may be present in many pesticides, which were also found to be more toxic than the formulations in rats [25] and bees [46]. Disruption of cell respiration and morphology in the NE-4C mouse cell line was previously shown for R Classic containing 15.5% of POEA [47], and for POEA in JEG3, HEK293, and HepG2 human cell lines, as well as for nine GBHs, for which the cytotoxicity was correlated to the concentration of ethoxylated co-formulants [8]. It was thus concluded that ethoxylated co-formulants are the ingredients responsible for human cell toxicity. Here we confirm this finding with 5 other GBHs and four other co-formulants, including non-ethoxylated co-formulants. Surprisingly, R WeatherMAX, which uses the “Transorb2 technology” (a plant cell penetrant system which was developed to bypass the problem of the toxicity of POEA), was the most toxic GBH among those tested, including those containing POEA. The only available information on this formulation is the presence of petroleum distillate. Such a petroleum distillate (solvent naphtha) was associated with reduced fertility and growth of newborn mice in laboratory tests [48]; further studies are needed in order to document the mechanism of its toxicity.

For the first time, we show endocrine disrupting effects for co-formulants of GBHs and for the GBH formulations tested. All co-formulants inhibited aromatase activity at concentrations 20%–67% below the NOEC (40%–240% below the toxicity threshold or LOEC). Aromatase was also inhibited by all formulations of GBH at levels 20%–200% below the NOEC, 40%–240% below the LOEC, and 33–400 times below the lowest recommended agricultural dilution. This was clearly due to co-formulants, since G alone did not significantly inhibit aromatase at these levels, but only at one-third of the agricultural dilution. GBHs were previously found to inhibit aromatase in human cells [15,37,38], but our current results demonstrate the involvement of co-formulants, not G alone, which was previously the general belief. 

These above cited studies were challenged by industry-linked scientists [28] in a review that was itself challenged [49,50]. The main claim was the non-suitability of *in vitro* models. In fact, cell cultures replace, whenever possible, animal experimentation [51]. Furthermore, our model may underestimate real toxicity, since cell lines are less sensitive than primary cells [16], and because a 24 h exposure does not anticipate possible bioaccumulation, and it is a necessary primary approach to understand mechanistic effects. 

Furthermore, endocrine-disrupting effects of GBHs were also evidenced *in vivo* on non-target species [26,27,52,53]. These effects, including alteration of sex steroid hormones, were observed at levels between 5–50 mg/kg body weight/day (ppm bw/d) of G (in formulation with co-formulants), while the doses of GBHs provoking aromatase inhibition are 25 ppm (Glyfos, corresponding to 9 ppm G with co-formulants), 50 ppm (all the other formulations except Medallon, 300 ppm). In a chronic rat feeding study, we recently evidenced endocrine disrupting effects of R on the estrogen/androgen balance starting at 0.1 ppb (corresponding to 4.5 10^−6^ ppm bw/d of G with co-formulants), triggering hormone-dependent mammary tumors and pituitary and sex hormone disruptions in females [54]. Our results indicate that all these effects are potentially related to co-formulants in formulations, especially if they are themselves endocrine disruptors. This was observed, for instance, for 4-n-nonylphenol or 4-n-octylphenol, widely used as surfactants and shown to inhibit the aromatase pathway [55]; but they were not compared to dAIs or commercial formulations.

Aromatase inhibition leading to a change in the balance of sexual steroids could partly explain most of the effects cited above and reviewed by Mesnage *et al.* [56]. Aromatase resides as a membrane-bound protein in the endoplasmic reticulum (ER) and membrane-disrupting effects of co-formulants are well established [6,8]. Perturbation of the mitochondrial membrane by several surfactants of GBH was shown to provoke a concentration-dependent decrease in steroid formation in hCG-stimulated MA-10 Leydig cells [57]. Decreases in progesterone production were explained by the inhibition of import, processing, and cholesterol transfer in the mitochondria. This was due to the loss of mitochondrial membrane potential resulting from penetration of co-formulants after an acute exposure to low doses of formulations of R, or to co-formulants alone. Ethoxylated non-ionic surfactants (related to the co-formulants POEA and POE-APE studied here) and nonylphenol polyethoxylates (NPEOs) were shown to interact with proteins and phospholipids in cell membranes, modifying the membranes’ structure and permeability [58]. This led to increased intracellular Ca^2+^ levels and changes in endoplasmic reticulum (ER) ultrastructure, activating the ER-stress signal pathway [59,60]. Thus, disruption of the ER membrane ultrastructure and function may explain the inhibition of aromatase activity or estrogen and androgen receptors [38]. Finally, G was previously shown to interact with the active site of aromatase at higher doses [37] and to inhibit aromatase at 3000 ppm. G was also reported to exert proliferative effects in human estrogen-dependent cells [61,62], and possible interactions with the estrogen receptors from 10^−12^ M in estrogen withdrawal conditions [62]. Co-formulants may promote a direct interaction between G and aromatase by increasing G intake by the cells. A direct interaction may also occur between co-formulants and the membrane-bound aromatase together with its electron donor cytochrome P450-reductase. GBH formulations contain other chemical co-formulants [54] that may also play a role. For example, although G alone did not significantly inhibit aromatase below 3000 ppm, the existence of other(s) untested chemical(s) in the formulations may change the effect of G on aromatase, or its threshold of action.

Most of the adverse (toxic and endocrine-disrupting) effects measured here with formulations of GBHs could be attributed to the co-formulants they contain, and none of the co-formulants tested here were found to be inert in human cells. Our results and others compiled in Mesnage *et al.* (2015) [56] show that the distinction between “active ingredient” and “inert compound” is a regulatory assertion with no toxicological ground. Indeed, the toxicity of co-formulants in pesticides is increasingly well documented [22,63,64,65]. High volumes of co-formulants (also called surfactants) are used; thus, they (or their breakdown products) can be found in the environment [66,67,68] and food [69,70,71]. All the honey, pollen, and wax samples monitored in a recent study were contaminated with high levels (up to 10 ppm) of nonylphenol polyethoxylates (NPEOs), a major class of surfactants in pesticides [72]. Their absorption by living beings [73] and placental transfer into serum and brain were evidenced [74]. A regulatory assessment claiming to investigate the safety of a formulation should take into account the toxicity of the co-formulants, which currently is poorly studied, with only their acute ocular and dermal properties being investigated [75]. This confusion between G and GBH underestimates the real toxicity and endocrine-disrupting properties of pesticides as sold, sprayed, and found in the environment, water and food. This has important regulatory consequences because the Acceptable Daily Intake (ADI) value is defined according to the threshold of toxicity calculated with the dAI alone. The ADI value does not take into account the co-formulants present in the formulations. 

## 5. Conclusions

Up to now, endocrine-disrupting effects of pesticides have been studied mostly based on tests on their declared active ingredient. Here we report for the first time that, below their toxicity thresholds, the co-formulants, generally classified as inerts and kept confidential, act as endocrine-disrupting chemicals at levels up to several hundred times below the level at which the declared active ingredient demonstrates the same activity. Glyphosate is never used alone, but always with its co-formulants. Thus the physiological effects of co-formulants should be more thoroughly tested and declared. We also recommend that the calculation of the ADI for pesticides should be based on toxicity tests of the commercial formulations rather than solely the declared active ingredient.

## Figures and Tables

**Table 1:** Toxicological and chemical characteristics of the glyphosate-based herbicides (GBHs), the co-formulants, and the declared active ingredient (dAI) investigated in this study. Contents in co-formulants and glyphosate (type of salt and equivalent in glyphosate acid) are indicated for the GBH formulations. LC_50_ (ppm) were calculated by nonlinear regression using asymmetric (5-parameters) equation with GraphPad Prism 5 after mitochondrial respiration inhibition measurement in JEG3 cells (MTT assay, see Figure 1). No Observed Effect Concentration (NOEC) and Lowest Observed Effect Concentration (LOEC, corresponding to the threshold of toxicity) were determined by MTT assay for each compound. They respectively correspond to the highest concentration without significant cytotoxic effect and to the lowest concentration with significant cytotoxic effect (in ppm). Can Canada, IPA isopropyl ammonium, G glyphosate, Hun Hungary.

	Products	Trade Name (Manufacturer, Country)	Declared Active Ingredient (dAI)	dAI (%)	Present in	(ppm)	NOEC	LOEC	LC50
**Co-formulants**	POEA	Emulson AG GPE 3SS (Lamberti, Ita)	Polyethoxylated tallow amine	100	Roundup Classic, Glyfos		3.0	3.5	3.9
	POEA/F	Emulson AG GPE 3/SSM (Lamberti, Ita)	Polyethoxylated tallow amine	70	Roundup Classic, Glyfos		4.0	4.5	4.7
	QAC	Emulson AG CB 30 (Lamberti, Ita)	Quaternary ammonium compound	30	other herbicides		35	50	58
	POE-APE	Rolfen Bio (Lamberti, Ita)	POE alkyl phosphate ether	70	other herbicides		150	200	222
	APG	Plantapon LGC (The Soap kitchen, UK)	Alkyl polyglucoside	28.5–34.0	Medallon Premium		200	400	421
			G salt of (g/L)	G (g/L)	Co-formulants (%)				
**Formulations**	RWMAX	Roundup WeatherMAX (Monsanto, Can)	Potassium (660)	540	Petroleum distillate /Transorb2		60	70	71
	Glyfos	Glyfos (Cheminova, Hun)	IPA (486)	360	9% POEA		75	85	86
	R Classic	Roundup Classic (Monsanto, Hun)	IPA (486)	360	15,5% POEA		75	80	89
	Kapazin	Kapazin (Arysta, Hun)	IPA (486)	360	C8-10 ethoxylated alcohol (<2 g/L), Triethylene glycol monobutyl ether (<2 g/L)		75	85	128
	Total	Total (Sinon Corporation, Hun)	IPA (486)	360	58.5% unknown surfactant		100	125	130
	Medallon	Medallon Premium (Syngenta, Hun)	diammonium (433)	360	10%–20% APG (150 g/L)		500	600	1268
**dAI**	G	Glyphosate isopropyl ammonium (Hun)	IPA (486)	360			3100	4600	7878

**Table 2:** Chemical structures of the co-formulants and declared active ingredient of glyphosate-based herbicides investigated in this study. CAS registry numbers are declared by the manufacturers or suppliers.

Chemical Structure	CAS RN *	Chemical Class of Substance Group/Substance Name
**Co-Formulants**		
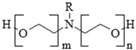	61791-26-2	polyethoxylated tallowamine (POEA) (R = C_14_–C_18_) (*n* + *m*= 2–28)
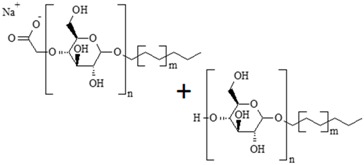	383178-66-3 + 110615-47-9	alkyl polyglucosides (APG) (*n* < 3, m = 3–6)
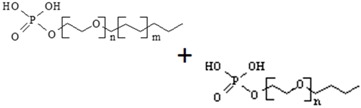	68130-47-2 + 50769-39-6	polyoxyethylene alkyl ether phosphates (POE-APE) (*n* = 6–10, m = 2–3)
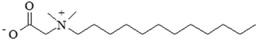	66455-29-6	quaternary ammonium compound (QAC)
**Active Ingredient**		
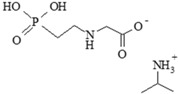	386411-94-0	isopropylamine salt of glyphosate

* Chemical Abstracts Registry Number.
